# A275 THE POTENTIAL MECHANISTIC ROLES OF SPHINGOLIPIDS IN HEALTHY FIRST-DEGREE RELATIVES OF PATIENTS WITH CROHN'S DISEASE

**DOI:** 10.1093/jcag/gwad061.275

**Published:** 2024-02-14

**Authors:** M Xue, S Lee, A Neustaeter, J SHAO, H Huynh, A Griffiths, D Turner, K Madsen, P Moayyedi, H Steinhart, A Bitton, D Mack, K Jacobson, M Ropeleski, M Cino, C Bernstein, R Panaccione, B Bressler, W Turpin, K Croitoru

**Affiliations:** lunenfeld-tanenbaum research institute, Mount Sinai Health System, Toronto, Toronto, ON, Canada; lunenfeld-tanenbaum research institute, Mount Sinai Health System, Toronto, Toronto, ON, Canada; lunenfeld-tanenbaum research institute, Mount Sinai Health System, Toronto, Toronto, ON, Canada; Statistics, University of Toronto, Toronto, ON, Canada; University of Alberta, Edmonton, AB, Canada; The Hospital for Sick Children, Toronto, ON, Canada; The Hebrew University of Jerusalem, Jerusalem, Israel; University of Alberta, Edmonton, AB, Canada; Farncombe Family Digestive Health Research Institute, Hamilton, ON, Canada; lunenfeld-tanenbaum research institute, Mount Sinai Health System, Toronto, Toronto, ON, Canada; McGill University Health Centre, Montreal, QC, Canada; Children’s Hospital of Eastern Ontario and University of Ottawa, Ottawa, ON, Canada; University of British Columbia, Vancouver, BC, Canada; Queen’s University, Kingston, ON, Canada; Statistics, University of Toronto, Toronto, ON, Canada; University of Manitoba Max Rady College of Medicine, Winnipeg, MB, Canada; University of Calgary, Calgary, AB, Canada; University of British Columbia, Vancouver, BC, Canada; lunenfeld-tanenbaum research institute, Mount Sinai Health System, Toronto, Toronto, ON, Canada; lunenfeld-tanenbaum research institute, Mount Sinai Health System, Toronto, Toronto, ON, Canada

## Abstract

**Background:**

Crohn's Disease(CD) features a complex etiology intertwining genetic, environmental, and immunological dimensions. Sphingolipids (SPs) are pivotal lipid molecules within cell membranes and various biological processes, including cell growth, apoptosis, and inflammatory responses. While our prior research identified SPs as significant risk factors for CD onset, their pre-clinical influence on CD risk biomarkers and potential role in pathogenesis remains unclear

**Aims:**

To identify the relationship between SPs and established CD risk factors

**Methods:**

We used samples from healthy first-degree relatives(FDRs), followed in a nested case-control cohort from the Crohn's Colitis Canada- Genes, Environment, Microbial(GEM) project, matching those who developed CD(n=77) with control FDRs(n=303) based on age, sex, follow-up time, and geographic location. 59 SPs were selected from serum metabolites measured via an untargeted metabolomics platform. We used partial Spearman correlation to identify relationships between SPs and CD risk factors: i)intestinal permeability via the urinary fractional excretion ratio of lactulose to mannitol(LMR); ii)gut subclinical inflammation using fecal calprotectin(FCP); iii)systemic inflammation with C-reactive protein(CRP); iv)microbiome composition through fecal 16S rRNA sequencing and v)serum protein profile using the Olink® Proximity Extension Assay platform. A two-sided *q*ampersand:003C0.05 was considered significant

**Results:**

We identified 38 positive correlations between CRP and SPs across various sub-pathways, including *Ceramides, Sphingomyelins* and *Hexosylceramides*(0.117≤rho≤0.342, 1.37×10^−09^≤*q*≤0.042). One SP *Ceramide(d18:1/16:0)* correlated positively with FCP(rho=0.201, *q*=0.012). Moreover, two SPs, *N-palmitoyl-sphingosine (d18:1/16:0)* and *glycosyl-N-(2-hydroxynervonoyl)-sphingosine (d18:1/24:1(2OH)),* correlationed with *Peptoclostridium* genus (rho=0.465 and -0.245, *q*=1.47×10^−16^ and 0.028 respectively). All SPs correlated with one or more proteins, most positively between *Sphingosine-1-phosphate* and *non-receptor tyrosine kinase*(rho=0.637, *q*=1.98 ×10^−36^) and most negatively between *sphingadienine* and *Chymotrypsin-C protein*(rho=-0.334, *q*=4.11×10^−8^). No significant correlations emerged between SPs and LMR

**Conclusions:**

We identified correlations between SPs and CD risk factors. The correlation with CRP suggests SPs might contribute to systemic inflammatory pathways related to CD. Moreover, correlations with the bacterial taxa highlight SPs' potential role in regulating microbial composition. Extensive correlations with proteins emphasize the pivotal impact of SPs on their function. This study may offer new insights into CD prevention

**Funding Agencies:**

CCC, CIHR

